# MiR-22 suppresses epithelial–mesenchymal transition in bladder cancer by inhibiting Snail and MAPK1/Slug/vimentin feedback loop

**DOI:** 10.1038/s41419-017-0206-1

**Published:** 2018-02-12

**Authors:** Mingjie Xu, Jiangfeng Li, Xiao Wang, Shuai Meng, Jiaying Shen, Song Wang, Xin Xu, Bo Xie, Ben Liu, Liping Xie

**Affiliations:** 1 Department of Urology, The First Affiliated Hospital, School of Medicine, Zhejiang University, Hangzhou, China; 2 Department of Urology, Zhejiang Provincial People’s Hospital, Hangzhou, China; 3 Program of Cancer Innovative Therapeutics, Division of Hepatobiliary and Pancreatic Surgery, Department of Surgery, The First Affiliated Hospital, School of Medicine, Zhejiang University, Hangzhou, China; 4 Department of Urology, TongDe Hospital of Zhejiang Province, Hangzhou, China

## Abstract

MicroRNAs (miRNAs) have been validated to play prominent roles in the occurrence and development of bladder cancer (BCa). MiR-22 was previously reported to act as a tumor suppressor or oncomiRNA in various types of cancer. However, its accurate expression, function, and mechanism in BCa remain unclear. Here, we find that miR-22 is frequently downregulated in BCa tissues compared with adjacent non-cancerous tissues. Overexpression of miR-22 significantly inhibits proliferation, migration, and invasion of BCa cells both in vitro and in vivo. Importantly, miR-22 is found to suppress cell proliferation/apoptosis by directly targeting MAPK1 (mitogen-activated protein kinase 1, ERK2) and inhibit cell motility by targeting both MAPK1 and Snail. Further statistical analysis shows that low-expression of MAPK1 or Snail is an independent prognostic factor for a better overall survival in patients with BCa (*n* = 401). Importantly, we describe an important regenerative feedback loop among vimentin, Slug and MAPK1 in BCa cells. MAPK1-induced Slug expression upregulates vimentin. Vimentin in turn activates MAPK1. By inhibiting Snail and MAPK1/Slug/vimentin feedback loop, miR-22 suppresses epithelial–mesenchymal transition (EMT) of BCa cells in vitro as well as in vivo. Taken together, this study reveals that miR-22 is critical to the proliferation, apoptosis and EMT progression in BCa cells. Targeting the pathway described here may be a novel approach for inhibiting proliferation and metastasis of BCa.

## Introduction

Bladder cancer (BCa) is the 9th most frequently diagnosed cancer worldwide. Although the mortality rate of bladder cancer tends to decrease, bladder cancer still ranks 13th in terms of death rate^1^. About one-third of BCa patients develop muscle-invasive or metastatic disease^2^. Muscle-invasive bladder cancer is highly heterogeneous in which approximately half of the patients are cured by surgery, while the other half progresses to the rapid disease progression^3^. Thus, improved understanding of the precise molecular mechanisms underlying BCa migration, invasion, and metastasis is urgently needed.

Epithelial–mesenchymal transition (EMT) is the molecular reprogramming and phenotypic changes characterizing the conversion of polarized immotile epithelial cells to motile mesenchymal cells^4^. Members of Snail family (Snail/Snail1 and Slug/Snail2) are critical inducers of EMT progression^5–7^. The expression of Snail is closely associated with cancer metastasis^8^. It has been reported that Snail is required for lymph node metastasis of human breast carcinoma MDA-MB-231 cells^9^. Slug was found to induce EMT progression by enhancing vimentin expression and migration in pre-malignant breast epithelial cells^10^. MAPK1 (mitogen-activated protein kinase 1, ERK2) is an important member of MAPK/ERK pathway and known to regulate the transcription of target genes both directly (by direct binding to the promoter region of the target gene)^11^ and indirectly (by regulating the activity or expression levels of transcription factors)^12^.

MicroRNAs (miRNAs) are short non-coding RNA molecules that usually repress gene expression by binding to the 3′-untranslated region (3′-UTR) of their target mRNAs^13^. Increasing evidence indicates that miRNAs have important roles in the formation of BCa^10,14^. Our group previously identified a series of miRNAs, including miR-409-3p^15^, miR-490-5p^16^, miR-576-3p^17^ and miR-433^18^ that are involved in the proliferation, migration, and invasion of BCa cells. MiR-22-3p (miR-22), primitively cloned from HeLa cells, is an evolutionarily-conserved gene located on chromosome 17p13^19^. In acute myeloid leukemia, miR-22 was revealed to target multiple oncogenes, including CRTC1, FLT3, and MYCBP; thus inhibiting the CREB and MYC pathways^20^. Recently, in colorectal cancer and gastric cancer, miR-22 has been reported to significantly inhibit EMT process and distant cancer metastasis by directly targeting member matrix metalloproteinase 14 and Snail^21^. However, some reported that miR-22 might act as an oncogene to promote proliferation, migration, and invasion of prostate and breast cancer^22,23^. Despite surging studies of the biogenesis and mechanisms of miR-22 were involved in the pathogenesis of diverse tumors, the accurate expression and mechanistic function of miR-22 in BCa remain unclear.

Here, we discovered that miR-22 is downregulated in BCa tissues. Both in vitro and in vivo studies showed that miR-22 is a critical suppressor to inhibit proliferation, invasion, and metastasis of BCa. Furthermore, we successfully demonstrated that miR-22 inhibits tumor invasion and metastasis by suppressing Snail and MAPK1. Importantly, we described a reciprocal regulation among MAPK1, Slug and vimentin.

## Results

### MiR-22 is downregulated in BCa

**Fig. 1:**
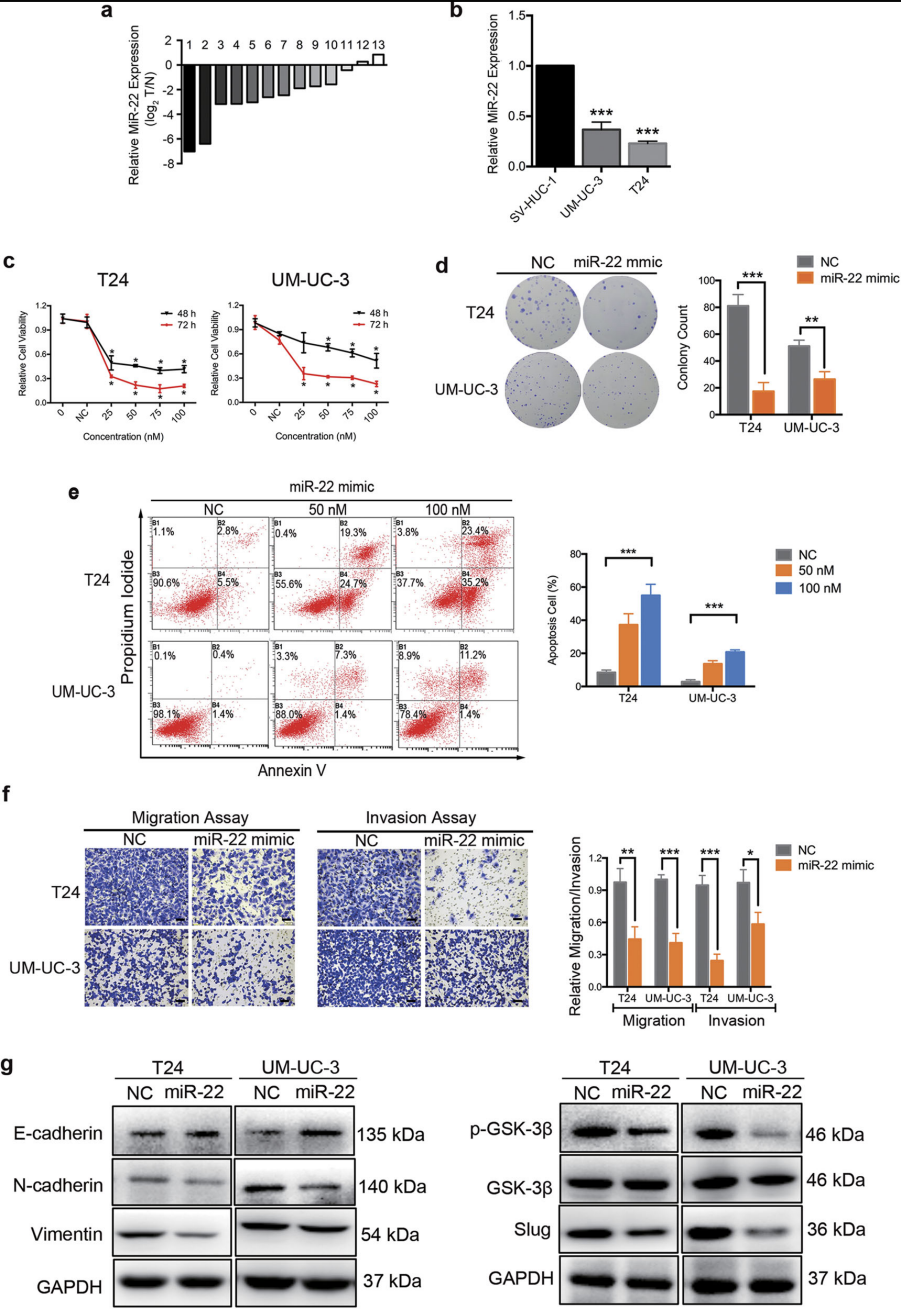
MiR-22 promotes apoptosis, inhibits proliferation and motility of BCa cells in vitro. **a** The relative expression levels of miR-22 in individual 13 pairs of BCa tissues were presented as the fold change of miR-22 referred to the corresponding adjacent normal tissues (T/N). **b** The miR-22 levels in two BCa cell lines (UM-UC-3 and T24) were detected by quantitative real-time PCR (qRT-PCR) and compared with non-tumor urothelial cell line SV-HUC-1. **c** Cell count kit-8 (CCK-8) assay. BCa cells were treated with 50 nM miR-22 mimics or mimic negative control (NC). The relative cell viability of the miR-22 treated groups was lower than that of NC treated groups (cell viability of 0 nM was regarded as 1.0), respectively. **d** Representative images of colony-formation assay. BCa cells were treated with 50 nM miR-22 mimics or NC for 7 days. The colony-formation rate was lower in miR-22 treated groups than that in NC treated groups. **e** Representative images of apoptosis analysis. BCa cells were treated with miR-22 mimics or NC for 48 h. The results showed that overexpression of miR-22 effectively induced BCa cell apoptosis. **f** Representative images of 24-h transwell assay. BCa cells were pretreated with miR-22 mimics or NC for 48 h. MiR-22 suppressed the migration and invasion rate of T24 and UM-UC-3 cells. **g** Representative images of western blotting assay. BCa cells were treated with miR-22 mimics or NC for 48 h. MiR-22 significantly regulated epithelial—mesenchymal transition (EMT)-related proteins in T24 and UM-UC-3 cells. Error bars represent the S.D. obtained from three independent experiments. *P < 0.05, **P < 0.01, ***P < 0.001. Scale bars = 100 μm

To evaluate the expression level of miR-22 in BCa, quantitative real-time PCR (qRT-PCR) was performed in 13 pairs of clinical BCa tissues and adjacent non-cancerous tissues (the clinical characteristics of the patients are shown in Supplementary Table 1). The expression level of miR-22 was frequently lower detected in tumor tissues than in non-tumor tissues (Fig. 1a, 11 out of 13 displayed a downregulation pattern). In two different urinary BCa lines (T24 and UM-UC-3), miR-22 was also less expressed in comparison with the non-tumor urothelial cell line SV-HUC-1 (Fig. 1b).

### MiR-22 inhibits proliferation and induces apoptosis of BCa cells in vitro

To assess the role of miR-22 in the regulation of BCa proliferation in vitro, T24 and UM-UC-3 cells were transfected with miR-22 mimics or mimic negative control (NC; Supplementary Figure 1a). MiR-22 inhibited the growth of BCa cells at different concentrations and time points (Fig. 1c). The 7-day colony-formation experiment showed that, compared to the NC treatment, miR-22 mimics suppressed the colony-forming ability of T24 and UM-UC-3 cells (Fig. 1d). Further, we observed that overexpression of miR-22 induced apoptosis in a dose-dependent manner (Fig. 1e), but had little influence on cell cycle of BCa cells (Supplementary Figure 1b). These results show that miR-22 significantly inhibits proliferation and promotes apoptosis of BCa cells.

### MiR-22 suppresses cell motility and EMT progression of BCa cells in vitro

**Fig. 2:**
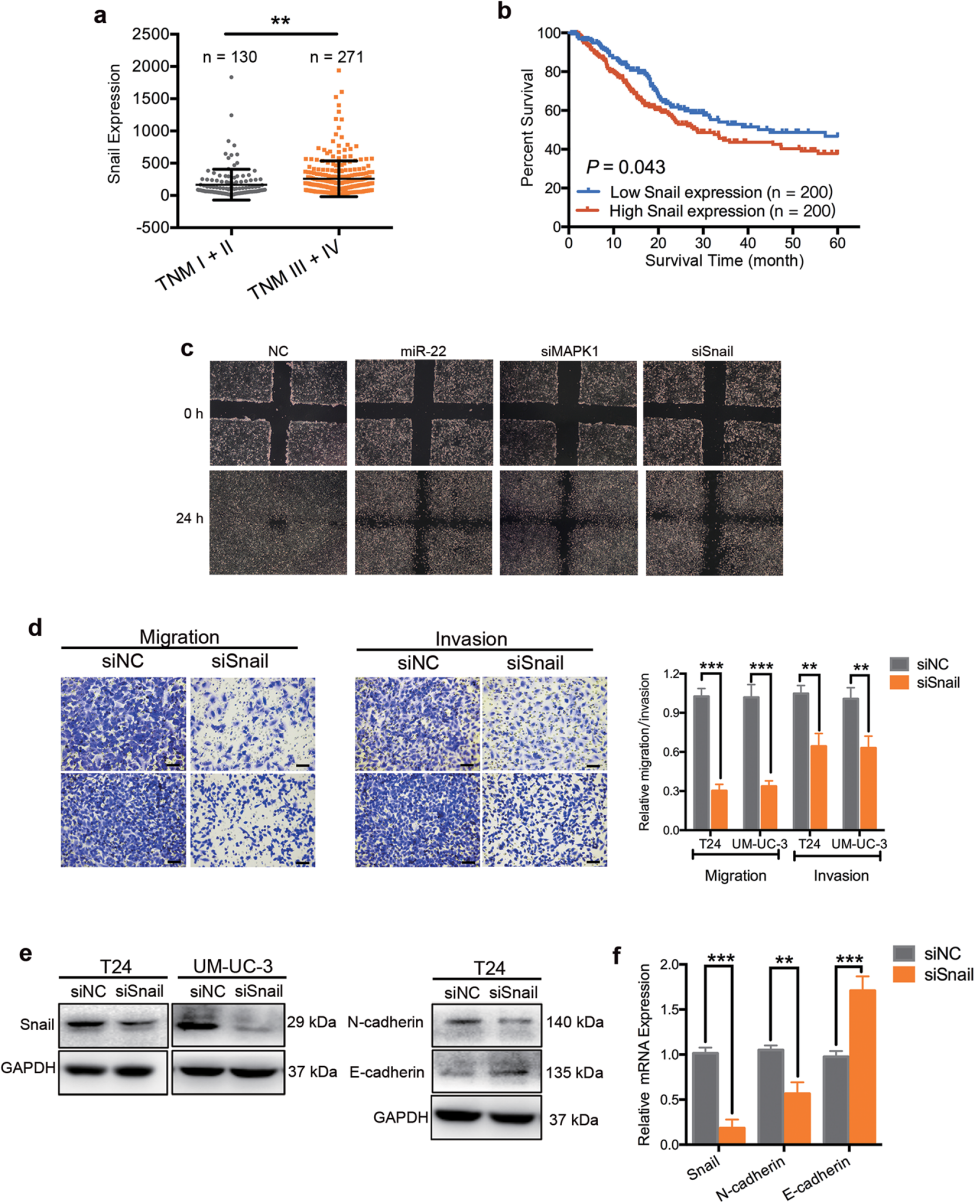
Knockdown of Snail inhibits BCa cell motility. **a** The relative expression levels of Snail mRNA of BCa cohort from TCGA with different TNM stages. **b** Kaplan–Meier survival curves of BCa cohort from TCGA with different Snail mRNA expression levels. **c** UM-UC-3 cells were pretreated with lipo2000 only (NC), miR-22 mimics, siMAPK1, or interfering RNA targeting human Snail mRNA (named siSnail) for 48 h, respectively. Wound-healing assay was performed with a 24-h recovery period. **d** Representative images of 24-h transwell assay. BCa cells were pretreated with siSnail or siNC for 48 h. Knockdown of Snail suppressed the migration and invasion rate of T24 and UM-UC-3 cells. **e** Representative images of the western blotting assay. BCa cells were treated with 50 nM siSnail or siNC for 48 h. Knockdown of Snail significantly regulated EMT-related proteins in T24 and UM-UC-3 cells. **f** qRT-PCR analysis. UM-UC-3 cells were treated as (**e**). Knockdown of Snail significantly regulated EMT-related mRNAs in UM-UC-3 cells. Error bars represent the S.D. obtained from three independent experiments. *P < 0.05, **P < 0.01, ***P < 0.001. Scale bars = 100 μm

To study the role of miR-22 in BCa migration and invasion, T24 and UM-UC-3 cells were transfected with miR-22 mimics or NC. The 24-h transwell assay revealed that, comparing to the NC treatment, miR-22 mimics effectively suppressed the migration and invasion capacity of both T24 and UM-UC-3 cells (Fig. 1f). In parallel, a wound-healing assay showed that ectopic expression of miR-22 in T24 and UM-UC-3 cells was associated with a retardation of wound closure compared with the NC treatment (Fig. 2c).

**Fig. 3:**
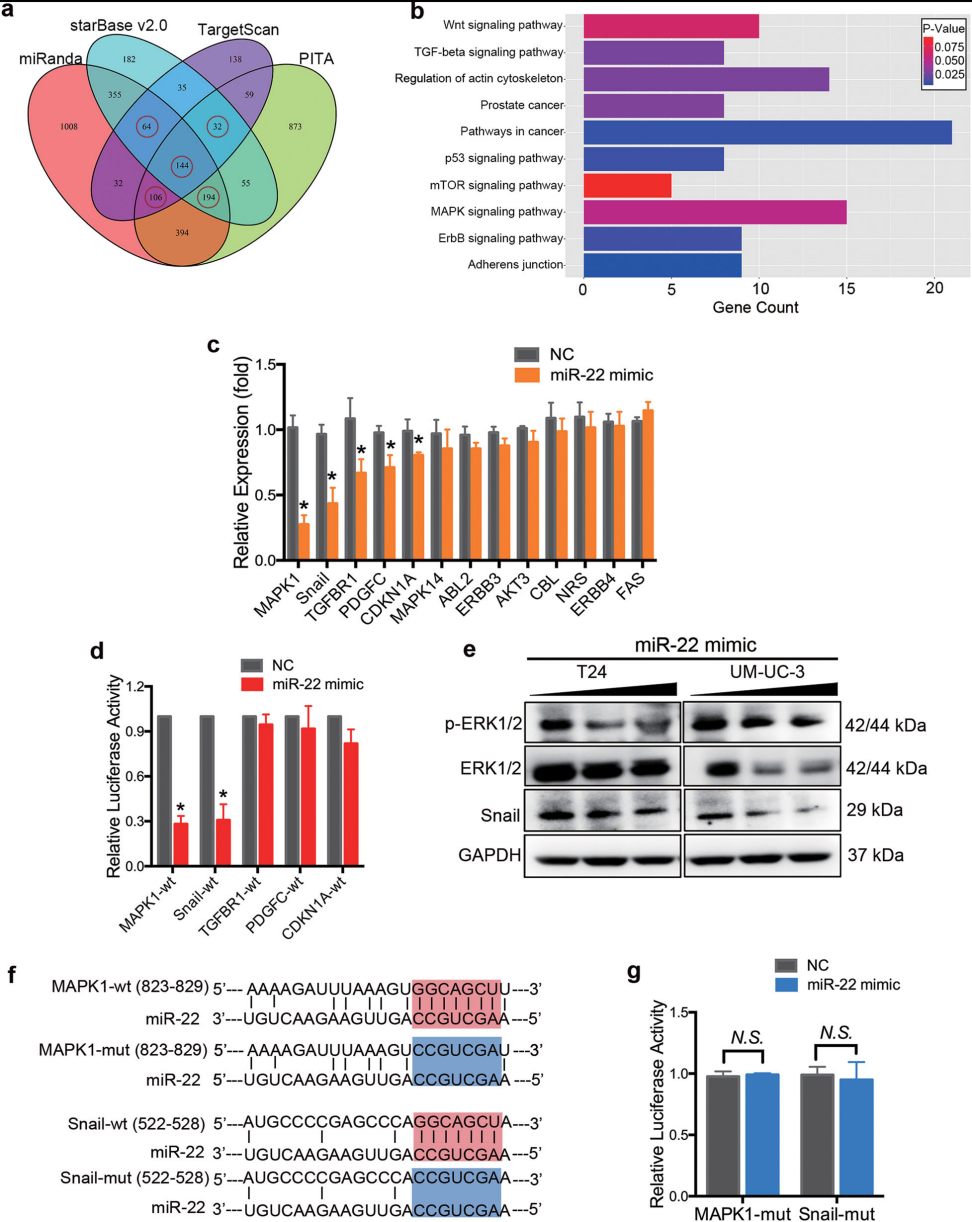
MAPK1 and Snail are direct targets of miR-22. **a** Venn diagrams of calculating the intersections of the four target prediction engines (miRanda, starBase v2.0, TargetScan and PITA; analyzed by R package VennDiagram). Potential miR-22 target genes were determined as being predicted by at least three of the four target prediction engines (*n* = 540). **b** Gene ontology enrichment analysis indicated that these 540 potential targets were involved in cancer and cell motility-related pathways. **c** qRT-PCR analysis. UM-UC-3 cells were treated with 50 nM miR-22 mimics or NC for 48 h. A decrease in the expression of MAPK1, Snail, TGFBR1, PDGFC and CDKN1A was detected in miR-22 transfected UM-UC-3 cells. **d** Dual-luciferase reporter assay. MiR-22 effectively suppressed the luciferase activity of vectors that carried 3′-UTRs of MAPK1 and Snail but not TGFBR1, PDGFC, and CDKN1A. **e** Representative images of the western blotting assay. MiR-22 (50 nM, 100 nM) significantly inhibited the expression of p-ERK2 (p-MAPK1), ERK2 (MAPK1) and Snail in T24 and UM-UC-3 cells. **f** Schematic representation of the miR-22 predicted binding sites in the 3′-UTRs of MAPK1/Snail mRNAs and 3′-UTR-mutated alignments. **g** Dual-luciferase reporter assay. The luciferase activities of the mutated vectors of MAPK1 and Snail were unaffected by the transfection of miR-22. Error bars represent the S.D. obtained from three independent experiments. *P < 0.05

In order to test the effect of miR-22 on EMT progression in BCa, the protein levels of EMT markers were analyzed by western blotting assay. Ectopic expression of miR-22 increased the expression of E-cadherin (an epithelial marker) but decreased N-cadherin, vimentin (mesenchymal markers), Snail, and Slug (EMT-related transcription factors). Additionally, overexpression of miR-22 also suppressed the phosphorylation of GSK-3β (EMT-related protein, phosphorylation is the inactive form of GSK-3β) (Figs. 1g and 3e). In conclusion, these results indicate that miR-22 suppresses cell motility and EMT phenotype of BCa cells in vitro.

### MAPK1 and Snail are direct target genes of miR-22

As the function of miRNAs in tumor development depends on their key target genes, it is important to identify the targets of miR-22. Potential targets were primarily determined as being predicted by at least three of the four target prediction engines (miRanda, starBase v2.0, TargetScan, and PITA)^24–27^ (Fig. 3a), and 540 genes were selected thereafter. Gene ontology (GO) and KEGG analysis^28,29^ indicated that these 540 potential targets were involved in cancer-related pathways (Fig. 3b and Supplementary Figure 1c). Among them, 47 genes were considered motility-related (data not shown). We identified 13 candidate target genes from these 47 genes and subsequently tested the specific mRNA expression levels after UM-UC-3 cells being transfected with miR-22 mimics. A decrease expression of MAPK1, Snail, TGFBR1, PDGFC, and CDKN1A was detected at mRNA level in miR-22-transfected UM-UC-3 cells (Fig. 3c). Dual-luciferase reporter assays were then performed to examine the direct interaction between miR-22 and the 3′-UTRs of these five genes (MAPK1, Snail, TGFBR1, PDGFC, CDKN1A). Among the five genes, overexpression of miR-22 only effectively decreased the relative luciferase activity of MAPK1 and Snail (Fig. 3d). In parallel, a western blotting assay showed a decrease in protein expression of MAPK1 and Snail in miR-22-transfected T24 and UM-UC-3 cells (Fig. 3e). To verify whether miR-22 directly binds to the 3′-UTRs of MAPK1 and Snail, we subsequently mutated the miR-22-targeting sites (Fig. 3f). The luciferase activity of the mutated vectors was not affected by the transfection of miR-22 (Fig. 3g). Taken together, these results reveal that MAPK1 and Snail are direct target genes of miR-22.

### Knockdown of MAPK1 induces apoptosis, inhibits proliferation, and cell motility in BCa cells

**Fig. 4:**
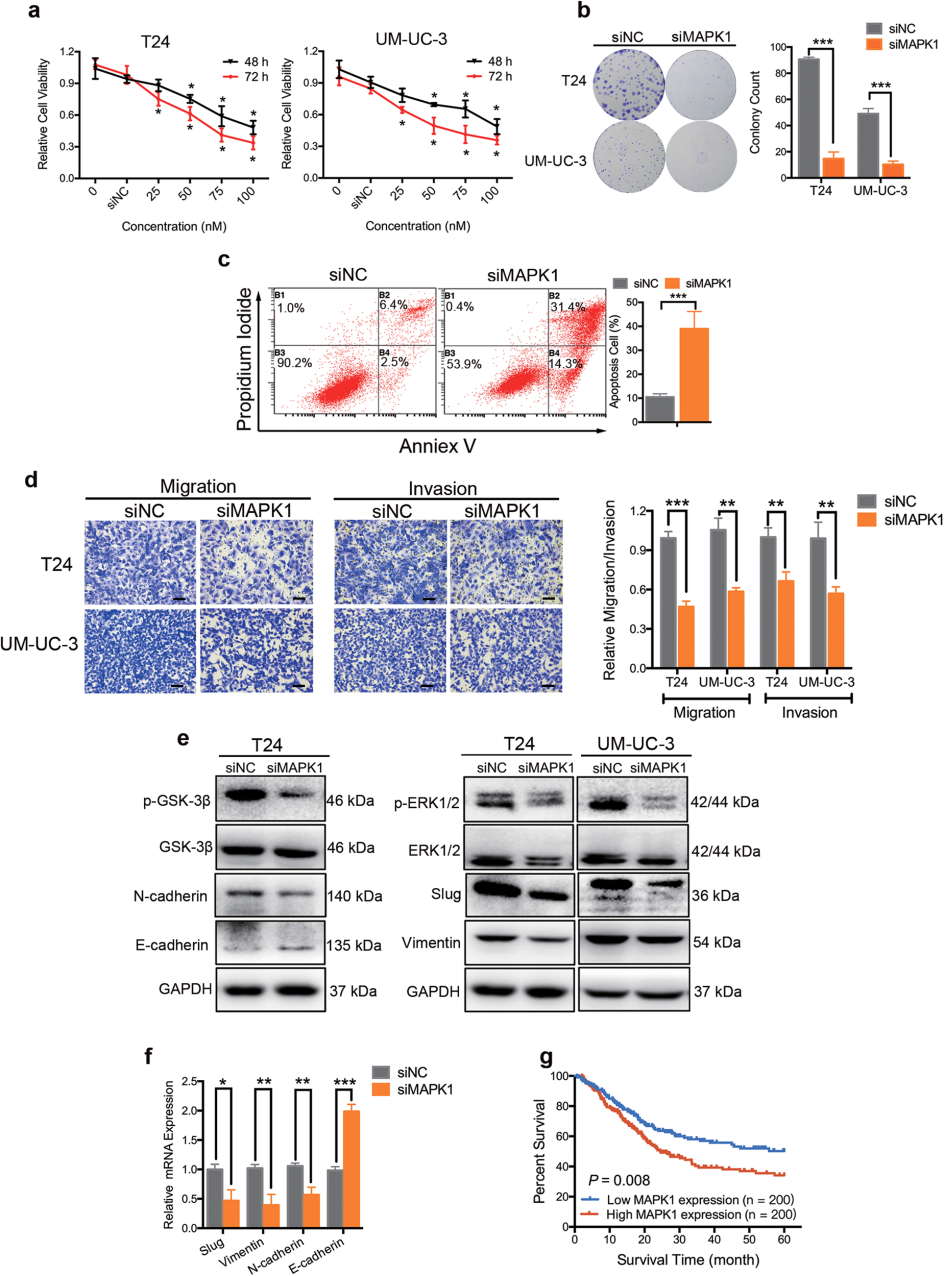
Knockdown of MAPK1 induces apoptosis, inhibits proliferation and motility of BCa cells. **a** CCK-8 assay. BCa cells were treated with 50 nM small interfering RNA targeting human MAPK1 mRNA (named siMAPK1) or siRNA negative control (siNC) for 48 h or 72 h. The relative cell viability of the siMAPK1 treated groups was lower than that of NC treated groups (cell viability of 0 nM was regarded as 1.0), respectively. **b** Representative images of colony-formation assay. BCa cells were treated with 50 nM siMAPK1 or siNC for 7 days. The colony-formation rate was lower for siMAPK1 treated groups compared with siNC treated groups. **c** Representative images of apoptosis analysis. T24 cells were treated with 50 nM siMAPK1 or siNC for 48 h. The results showed that silencing of MAPK1 effectively induced T24 cell apoptosis. **d** Representative images of the 24-h transwell assay. BCa cells were pretreated with 50 nM siMAPK1 or siNC for 48 h. The results showed that knockdown of MAPK1 suppressed the migration and invasion rate of T24 and UM-UC-3 cells. **e** Representative images of the western blotting assay. BCa cells were treated with 50 nM siMAPK1 or siNC for 48 h. Knockdown of MAPK1 significantly regulated EMT-related proteins in BCa. **f** qRT-PCR analysis. UM-UC-3 cells were treated as (**e**). Knockdown of MAPK1 significantly regulated EMT-related mRNAs in UM-UC-3 cells. **g** Kaplan–Meier survival curves of BCa cohort from TCGA with different MAPK1 expression levels. Error bars represent the S.D. obtained from three independent experiments. *P < 0.05, **P < 0.01, ***P < 0.001. Scale bars = 100 μm

To evaluate clinical significance of MAPK1 (ERK2) expression, we studied the related information of a BCa cohort from The Cancer Genome Atlas (TCGA; the clinical characteristics of the patients are shown in Supplementary Table 2; *n* = 401). Kaplan–Meier survival analysis revealed that BCa patients with low expression of MAPK1 had a better overall survival compared to those with high expression of MAPK1 (Fig. 4g). Thus, high MAPK1 expression may act as an indicator of poor prognosis in BCa. To assess the role of MAPK1 in the regulation of BCa proliferation in vitro, T24 and UM-UC-3 cells were knockdown of MAPK1 by specific small interfering RNA (siMAPK1). The CCK-8 assay revealed that the decrease of MAPK1 inhibited the growth of T24 and UM-UC-3 cells at different concentrations and time points (Fig. 4a). The colony-formation capacity of siMAPK1-transfected cells was much lower than that of cells transfected with siRNA negative control (siNC; Fig. 4b). We also observed that silencing of MAPK1 effectively induced apoptosis (Fig. 4c and Supplementary Figure 2a) and arrested the cell cycle of BCa cells (Supplementary Figure 2b). Next, we studied the role of MAPK1 in the motility of BCa cells. The wound-healing assay indicated that the downregulation of MAPK1 in BCa cells caused a retardation of wound closure compared with the NC treatment (Fig. 2c). The 24-h transwell assay revealed that migration and invasion capability were also inhibited in siMAPK1-transfected UM-UC-3 and T24 cells (Fig. 4d). In conclusion, these data reflect that downregulation of MAPK1 suppresses proliferation and motility of BCa cells.

Recently, increasing evidence has indicated that MAPK1 is involved in EMT induction^30,31^ and cell migration, and invasion in cancer cells^32,33^. In this study, western blotting assay and qRT-PCR assay showed that knocking down of MAPK1 increased the expression of E-cadherin but decreased N-cadherin, vimentin, and Slug. Moreover, silencing of MAPK1 also suppressed the phosphorylation of GSK-3β (Figs. 4e, f). Taken together, silencing of MAPK1 represents a novel approach for inducing apoptosis, inhibiting proliferation and motility of BCa cells.

### Knockdown of Snail inhibits BCa cell motility

To evaluate the relationship between Snail expression and BCa patients’ survival, we researched the corresponding information of a BCa cohort from TCGA (*n* = 401). The expression of Snail was lower detected in tumor tissues from BCa patients at early stages (tumor node metastasis (TNM) stages I and II) than in tumor tissues from BCa patients at advanced stages (TNM stages III and IV) (Fig. 2a). Furthermore, Kaplan–Meier survival analysis revealed that BCa patients with low expression of Snail had a better overall survival compared to those with high expression of Snail (Fig. 2b). Thus, high Snail expression may act as an indicator of poor prognosis in BCa. To assess the role of Snail in the regulation of BCa proliferation and apoptosis in vitro, T24 and UM-UC-3 cells were knocked down of Snail by siRNA (siSnail). The CCK-8 assay revealed that knocked down of Snail did not affect the growth of T24 and UM-UC-3 cells at different concentrations and time points (Supplementary Figure 2c). Silencing of Snail also had little influence on apoptosis and cell cycle of BCa cells (Supplementary Figure 2d and data not shown). Next, we studied the role of Snail in the motility of BCa cells. A wound-healing assay indicated that the downregulation of Snail in BCa cells caused a retardation of wound closure compared with the control treatment (Fig. 2c). The 24-h transwell assay revealed that migration and invasion ability were also being inhibited in siSnail-transfected UM-UC-3 and T24 cells (Fig. 2d). The western blotting assay and qRT-PCR assay showed that the knocking down of Snail increased the expression of E-cadherin but decreased N-cadherin (Figs. 2e, f), thus inhibited the EMT progression. Taken together, these results support that downregulation of Snail inhibits motility of BCa cells.

### MiR-22 controls EMT by inhibiting MAPK1/Slug/vimentin feedback loop and Snail

**Fig. 5:**
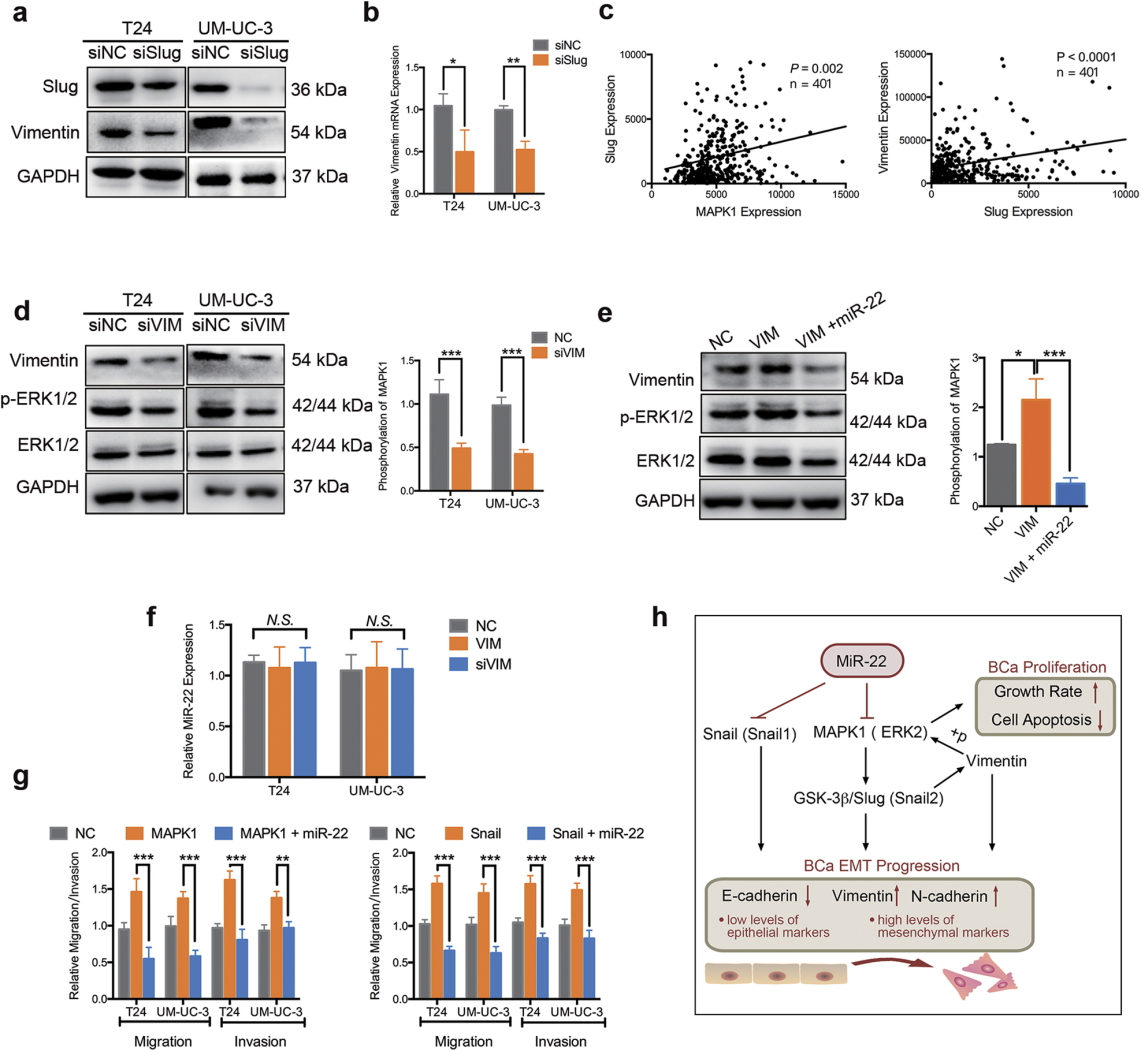
MiR-22 controls EMT by suppressing Snail and MAPK1/Slug/vimentin feedback loop. **a** Representative images of the western blotting assay. BCa cells were treated with 50 nM interfering RNA targeting human Slug mRNA (named siSlug) or siNC for 48 h. Knockdown of Slug inhibited vimentin protein expression in T24 and UM-UC-3 cells. **b** qRT-PCR assay. BCa cells were treated as (**a**). Knockdown of Slug inhibited vimentin mRNA expression in T24 and UM-UC-3 cells. **c** Statistical analysis revealed a positive correlation between the expression levels of slug and MAPK1 (left) or vimentin (right) mRNA in TCGA’s BCa cohort (*n* = 401). **d** Representative images of the western blotting assay. BCa cells were treated with 50 nM interfering RNA targeting human vimentin mRNA (named siVIM) or siNC for 48 h. Knockdown of vimentin suppressed phosphorylation of ERK2 (MAPK1), but did not affect expression levels of total ERK2 in T24 and UM-UC-3 cells. **e** Representative images of the western blotting assay. UM-UC-3 cells were transfected with lipo2000 only (NC), pc-DNA3.1- vimentin (VIM) or co-transfected VIM and miR-22 for 48 h. Overexpression of vimentin increased phosphorylation of ERK2 (MAPK1), but did not affect expression levels of total ERK2. MiR-22 mimic reversed the vimentin-induced ERK2 phosphorylation. **f** Representative images of the qRT-PCR assay. BCa cells were transfected with lipo2000 only (NC), VIM or siVIM for 48 h. Neither overexpression nor knocking down of vimentin affected the expression level of miR-22. **g** The 24-h transwell assay. BCa cells were transfected with lipo2000 only (NC), pc-DNA3.1- vimentin /MAPK1 (VIM/MAPK1) or co-transfected VIM/MAPK1 and miR-22. MiR-22 reversed migration and invasion that induced by MAPK1 (left) or Snail (right) overexpression in BCa cells. **h** A schematic diagram of the mechanisms under miR-22-mediated preoliferation, apoptosis and EMT regulation in BCa. Error bars represent the S.D. obtained from three independent experiments. *P < 0.05, **P < 0.01, ***P < 0.001

Previous studies revealed that overexpression of MAPK1 induced the transcriptional activity of Slug (Snail2), thus unregulated the expression of vimentin^34^. In BCa cells, we found that knocking down of MAPK1 suppressed the expression of Slug and vimentin (Figs. 4e, f). Silencing the expression of Slug by specific siRNA (siSlug) significantly suppressed vimentin at both protein and mRNA levels (Figs. 5a, b). We further found a positive correlation between the expression levels of Slug and MAPK1 or vimentin mRNA in TCGA’s BCa cohort (*n* = 401, Fig. 5c). These results indicated that Slug might act as a scaffold in MAPK1-induced vimentin expression in BCa cells. To assess the role of vimentin in the regulation of MAPK1 expression, BCa cells were knocked down of vimentin by specific siRNA (siVIM) or overexpression of vimentin by pcDNA3.1-vimentin (VIM). The western blotting assay revealed that silencing of vimentin suppressed phosphorylation of ERK2, but did not affect the expression levels of total ERK2. Besides, overexpression of vimentin increased the phosphorylation of ERK2 (Figs. 5d, e). However, silencing or overexpression of vimentin had little influence on the expression of miR-22 in BCa cells (Fig. 5f). Taken together, these data suggest that vimentin can in turn active MAPK1, thus forms a MAPK1/Slug/vimentin feedback loop in BCa cells.

To evaluate the effect of miR-22 on MAPK1/Slug/vimentin feedback loop, BCa cells were transfected with miR-22 mimics or NC. Should be noted, this vimentin-induced MAPK1 phosphorylation was abolished by miR-22 transfection in UM-UC-3 cells (Fig. 5e). The 24-h transwell assay revealed that MAPK1 or Snail-induced migration and invasion of BCa cells were effectively reversed by miR-22 overexpression (Fig. 5g). Conclusively, in BCa cells, miR-22 plays an important role in controlling of Snail-induced EMT progression and MAPK1/Slug/vimentin feedback loop (Fig. 5h).

### Overexpression of miR-22 inhibits proliferation and EMT of BCa cells in vivo

**Fig. 6:**
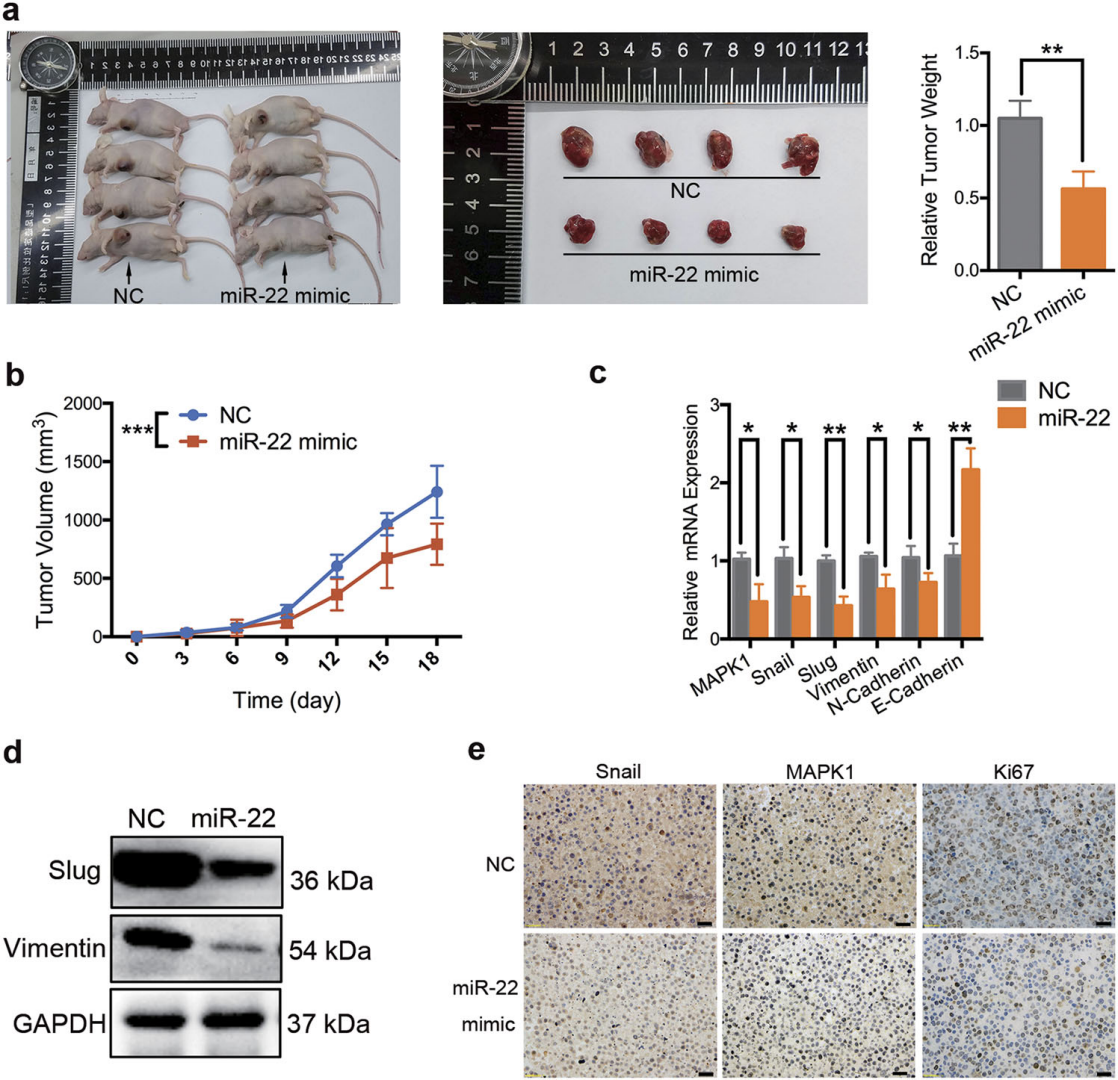
Overexpression of miR-22 inhibits proliferation and EMT of BCa in vivo. UM-UC-3 cells transfected with miR-22 mimic or nonsense dsRNA (NC) were implanted into nude mice. **a** When the animals were terminated at day 18, tumors of different groups were harvested and weighed. **b** Tumor volume was measured every 3 days. **c** qRT-PCR assay of tumor tissue that harvested from nude mice. The mRNA expression levels of Snail, MAPK1, Slug, vimentin and N-Cadherin were lower detected while E-Cadherin was higher detected in the miR-22 group compared with the NC group. **d** Representative images of the tumor tissue western blotting assay. The protein expression levels of Slug and vimentin were lower detected in the miR-22 treated group compared with the NC group. **e** Representative images of immunohistochemical staining (IHC) analysis. The protein expression levels of Snail, MAPK1, and Ki67 were lower detected in the miR-22 group compared with the NC group. Error bars represent the S.D. obtained from three independent experiments. *P < 0.05, **P < 0.01, ***P < 0.001. Scale bars = 100 μm

To further explore the effect of miR-22 on BCa in vivo, nude mice were implanted with UM-UC-3 cells and injected intratumorally with 30 μg of Lipofectamine 2000-encapsulated miR-22 mimic or nonsense dsRNA (NC) every 3 days. When the mice were terminated at day 18, tumors of different groups were harvested and weighed. The tumor xenograft studies revealed that the volumes of the tumors resulting from miR-22 mimic treatment group (miR-22 group) were significantly smaller than those resulting from NC treatment group (NC group) (Figs. 6a, b). In agreement with the tumor volumes, the weights of tumors from the miR-22 group were lower than that from the NC group (Fig. 6a). The qRT-PCR analysis confirmed that miR-22 expression levels in tumor tissues from the miR-22 group were increased compared with that in the NC group (Supplementary Figure 2e). Moreover, mRNA expression levels of miR-22-targeting genes and related downstream genes, MAPK1, Snail, Slug, N-cadherin, and vimentin were lower detected while E-cadherin were higher detected in tumor tissues from the miR-22 group compared to that in the NC group (Fig. 6c). In parallel, the western blotting or immunohistochemical staining (IHC) analysis revealed that the protein expression levels of MAPK1, Snail, Slug, and vimentin were lower in the tumor tissues from the miR-22 group than that in the NC group (Figs. 6d, e). Moreover, Ki67, a protein strictly associated with cell proliferation, was detected at lower levels in tumor tissues from the miR-22 group compared to that in the NC group (Fig. 6e). In conclusion, these data suggest that miR-22 effectively inhibits proliferation and EMT progression of BCa in vivo.

## Discussion

The expression of miR-22 was found downregulated in gastric cancer^21^, acute myeloid leukemia (AML)^20^, and esophageal squamous cell carcinoma^35^. The mechanisms under the loss of miR-22 are still poorly understood. It was reported that the downregulation of miR-22 in AML was caused by TET1/GFI1/EZH2/SIN3A mediated epigenetic repression and/or DNA copy-number loss^20^. In this study, qRT-PCR validation results showed that the expression of miR-22 was significantly lower in BCa tissues compared to that in adjacent non-cancerous tissues.

Increasing findings have documented a fascinating and normally ignored mechanism of miR-22 with reference to the regulation of cancer proliferation and EMT. However, conflicting results have been reported. For example, miR-22 promoted breast cancer proliferation, migration, and invasion by silencing acetylase TIP60^36^. Su Jung Song et al. found miR-22 as a crucial epigenetic modifier and promoter of EMT and breast cancer stemness towards metastasis^23^. However, miR-22 was found to induce p53 expression and concurrently target SIRT1, CDK6, and Sp1 to activate pRb signaling pathway; thereby hastening senescence, inhibiting cellular growth, invasion, and metastasis in cervical cancer and breast cancer^37^. Moreover, miR-22 suppressed EMT process and cancer distant metastasis by directly targeting TIAM1 (T-cell lymphoma invasion and metastasis 1) and SIRT1 in colorectal cancer and renal cell carcinoma, respectively^38,39^. Given the fact that miR-22 could directly target either proliferation or EMT-associated tumor suppressors or oncogenes to suppress or induce proliferation and metastasis, it is important to clarify the accurate expression and mechanistic function of miR-22 in different cancer types. In our present study, gain-of-function analyses showed that miR-22 induces apoptosis, suppresses the proliferation, migration, and invasion of BCa cells in vitro (Figs. 1c–f). The western blotting assay revealed that ectopic expression of miR-22 increased the expression of E-cadherin but decreased N-cadherin, vimentin, Snail, Slug, and phosphorylation of GSK-3β. These results revealed that miR-22 suppressed the EMT phenotype of BCa (Fig. 1g). Furthermore, a tumor xenograft mouse model verified that miR-22 inhibited proliferation and EMT progression of UM-UC-3 cells in vivo (Fig. 6). Taken together, these results indicate that miR-22 acts as an important tumor suppressor in BCa cells.

More importantly, our results have established MAPK1 (ERK2) and Snail as direct functional effectors of miR-22 in BCa. MiR-22 was previously reported to inhibit MAPK/ERK pathway indirectly^40,41^. By dual-luciferase reporter assays and western blotting analysis, we are the first to identify that miR-22 directly binds to 3′-UTR of MAPK1 mRNA and suppresses its expression (Fig. 3). MAPK/ERK signaling modulates epigenome to drive EMT^42^. Silencing of ERK/MEK reverses miR-21-mediated EMT in breast cancer cells^43^. Recent evidence has highlighted that ERK2 via DEF motif targets was sufficient to induce EMT^31^. In the present study, we found that high expression level of MAPK1 was correlated with a poor survival in BCa patients. Silencing of MAPK1 significantly promoted apoptosis, inhibited proliferation, migration, and invasion capability of BCa cells in vitro (Fig. 4). In neurons, vimentin fragments interact with ERK and support the translocation of active ERK in response to injury^44^. Reetta Virtakoivu et al. recently identified that vimentin functioned as an important and central EMT signaling scaffold supporting ERK activity in breast cancer cells^34^. We identified that MAPK1/Slug/vimentin were co-expressed in BCa cells (Fig. 5c). By western blotting assay and qRT-PCR analysis, we discovered that the knocking down of ERK2 inhibited vimentin expression via Slug and that vimentin facilitated ERK phosphorylation (though this remains to be formally shown; Fig. 5). Consistent with previous findings, we confirmed an important regulatory MAPK1/Slug/vimentin interaction facilitating MAPK1 phosphorylation with Slug at a novel site in BCa. It is reported that high Snail expression in superficial bladder tumors is a strong predictor of tumor recurrence and could be used to improve risk stratification and prognostication^45^. We found that the expression of Snail was, in fact, lower in tumor tissues from patients at early stages than in tumor tissues from patients at advanced stages. Likewise, BCa patients with low expression of Snail had a better overall survival compared with those with high expression of Snail. Snail was previously reported as a target of miR-22 in gastric cancer^21^. Our results confirmed this direct targeting in BCa cells by dual-luciferase reporter assays and western blotting analysis (Fig. 2). Further, we found that miR-22 overexpression effectively reversed both MAPK1- and Snail-induced migration and invasion in BCa cells (Fig. 5g). Thus, miR-22 is presented as a crucial tumor suppressor that controls MAPK1/Slug/vimentin feedback loop and represses Snail expression in BCa cells. Finally, it should be noted that a single miRNA could regulate the mRNA transcripts of hundreds of target genes. We cannot exclude the possibility that signaling pathways mediated by other targets, apart from Snail and MAPK1, may have a role in miR-22-mediated inhibition of EMT^36,39,42,46^.

In conclusion, we report the following findings: (i) MiR-22 functions as a tumor suppressor in BCa cells. (ii) MAPK1 and Snail are direct target genes of miR-22; (iii) both MAPK1 and Snail expression are independent prognostic factors for overall survival in patients with BCa; (iv) there is an interaction among vimentin, Slug and MAPK1 in BCa cells, which promotes MAPK1 activation and enhances vimentin expression; (v) by inhibiting Snail and MAPK1/Slug/vimentin feedback loop, miR-22 induces apoptosis, suppresses proliferation and EMT progression in BCa cells. Our study underscores the crucial role of miR-22 in BCa progression. We expect that our findings on miR-22-related proliferation inhibition and EMT repression will provide useful information for the development of more effective and promising therapies against BCa.

## Materials and methods

### Cell lines and cell culture

The human BCa cell lines T24, UM-UC-3, as well as one normal bladder cell line SV-HUC-1, were purchased from the Shanghai Institute of Cell Biology, Shanghai, China. These cell lines were maintained in Roswell Park Memorial Institute 1640 medium (RPMI1640; Gibco, Carlsbad, CA, USA) with 10% fetal bovine serum (FBS; Biological Industries, Cromwell, CT, USA), under a humidified atmosphere of 5% CO_2_ at 37 °C. The cell culture medium was changed every 2–3 days, and the cells were passaged with 0.25% trypsin-EDTA (Gibco) and grown to 90% confluence.

### Animal experiments

Male BALB/c-nude mice were purchased from the Shanghai Experimental Animal Center, Chinese Academy of Sciences, Shanghai, China. Each mouse was 4 weeks old, weighing 18~20 g. UM-UC-3 cells (1 × 10^6^ in 50 μl PBS) were injected subcutaneously into the right axilla of each mouse. When tumors could be observed, 12 mice were randomized into 2 groups. Then those mice were injected intratumorally with 30 μg of Lipofectamine 2000-encapsulated miR-22 mimic or nonsense dsRNA (NC) every 3 days for 18 days. Tumor size was monitored and evaluated every 3 days by measuring the two perpendicular diameters. The volume of the tumor was calculated with the formula V = (width^2^ × length × π/6). For each group, the highest and lowest tumor volumes were dropped before calculation. At the conclusion of the experiment, the mice were euthanized. The tumors were removed and fixed in 4% formalin and paraffin-embedded.

### TCGA database

TCGA is available from the website of the Cancer Genomics Browser of the University of California, Santa Cruz (https://genome-cancer.ucsc.edu/). MAPK1 and Snail mRNA expression and miRNA-sequencing level 3 data in BCa patients were extracted from TCGA’s data portal. Only patients with fully characterized tumors, TNM stage, overall survival, complete miRNA information were included. In total, data on 401 BCa patients with detailed miR-22, MAPK1 and Snail expression were collected from TCGA’s data portal. Read counts for both mRNAs and miRNAs were used as input for survival analysis with the R package edgeR^47,48^. Kaplan–Meier plots for MAPK1 or Snail expression in association with overall survival were calculated with the R program. Patients were split into high and low expression groups based on the median expression of MAPK1 or Snail.

### GO and pathway analysis

To inspect the function of miR-22 potential target genes, we included the experimentally validated targets and performed GO analysis using GO enrichment analysis with a *P*-value threshold of 0.005. Functional annotation analysis was conducted using DAVID tools (http://david.abcc.ncifcrf.gov) to query KEGG pathways enriched with predicted miRNA targets. The analyses were conducted using the “fuzzy clustering algorithm” in order to reduce the redundancy among functionally related pathways that share similar target genes. Terms with Benjamini-corrected enrichment *P*-values < 0.01 and FDR < 0.05 were considered.

### Reagents and transfection

The hsa-miR-22-3p mimic (named miR-22 mimic; miRBase accession MIMAT0000077; sense: 5′-AAGCUGCCAGUUGAAGAACUGU-3′) and the negative control duplex (named NC, sense: 5′-ACUACUGAGUGACAGUAGA-3′) with no significant homology to any known human sequences were used for gain-of-function studies. The small interfering RNA targeting human MAPK1 mRNA (named siMAPK1), Snail mRNA (named siSnail), and vimentin mRNA (named siVIM) were used for the RNA interference study (Supplementary Table 4). The RNA duplexes were chemically synthesized by GenePharma, Shanghai, China. Oligonucleotide transfection was performed using Lipofectamine 2000 reagents (Invitrogen, Carlsbad, CA, USA) in accordance with the manufacturer’s protocol.

### Dual-luciferase reporter assay

Oligonucleotide pairs that contained the desired miR-22 target region or mutant target region were designed and ordered from Sangon, Shanghai, China. After annealing, these double-stranded segments were inserted into pmirGLO Dual-Luciferase miRNA Target Expression Vector (Promega, Madison, WI, USA), between the SacI and SalI sites. The insertions were verified by sequencing. Dual-luciferase assays were performed using 1 × 10^4^ UM-UC-3 cells per well in a 96-well plate (Corning/Costar, Acton, MA, USA). After the cells attached for 8 h, they were cotransfected with 50 ng of respective reporter constructs with either 50 nM of miRNA mimics or control miRNA. After 48 h, a Reporter Assay System Kit (Promega, Beijing, China) was used to measure the luciferase activity. There were three replicates for each transfectant. Firefly luciferase activity was normalized to constitutive Renilla luciferase activity.

### Cell growth/cell viability assay (Cell Count Kit-8 assay)

T24 or UM-UC-3 cells were plated in 96-well plates with ~4 × 10^3^ cells per well. After overnight incubation, the cells were transfected with the RNA duplex (miR-22, siMAPK1, siSnail, or NC) for 2–3 days with concentrations ranging from 25 to 100 nM. At different time points, the medium was removed and WST-8 (Dojindo Laboratories, Kumamoto, Japan) was added to each well. After the 96-well plate was incubated at 37 °C for 1 h, the absorbance of the solution was measured spectrophotometrically at 450 nm with an MRX II absorbance reader (Dynex Technologies, Chantilly, VA, USA).

### Colony-formation assay

Cells with different treatment were trypsinized to a single cell suspension 24 h after transfection with 2′-O-methyl-modified duplexes (50 nM). Next, the cells were seeded in six-well plates (500 cells per well) and maintained under standard culture conditions for 2 weeks. Colony counts were performed after the colonies were fixed with absolute methanol and stained with 0.1% crystal violet.

### Wound-healing assays

After transfection, the cells with different treatment were grown to 100% confluence in six-well plates. A micropipette tip was used to make a cross wound and wound healing was observed after 24 h. Photographs were taken under phase-contrast microscopy (Olympus, Tokyo, Japan) immediately or 24 h after wounding.

### Cell migration and invasion assay

The cell migration and invasion assay were performed using transwell chambers (Millipore, Boston, MA, USA). For the invasion assay, the inserts were coated with Matrigel (BD Bioscience, Franklin Lakes, NJ, USA) on the upper surface. After transfection, 8 × 10^4^ cells were suspended in 0.2 ml serum-free medium and added to the inserts. Then, 0.6 ml RPMI-1640 medium with 10% FBS was added to the lower compartment as a chemoattractant. After incubation at 37 °C for 24 h, the cells on the upper surface of the membrane were carefully removed using a cotton swab and cells on the lower surface were fixed with 100% methanol and stained with 0.1% crystal violet. Five visual fields of 200× magnification of each insert were randomly selected and counted under a light microscope (Olympus).

### Vector constructs

Expression vectors encoding vimentin were constructed by cloning the open reading frames into the pcDNA 3.1 vector (Invitrogen) between the HindIII and EcoRI sites for expression driven by the CMV promoter (pcDNA3.1-vimentin).

Detailed methods are described in the Supplementary Materials, including IHC analysis, Annexin V and propidium iodide staining analysis, flow cytometry analysis of cell cycle, RNA extraction and real-time quantitative PCR (qRT-PCR) analysis, western blotting assay, statistical analysis, and ethics statement.

## Electronic supplementary material

Supplementary Figure 1

Supplementary Figure 2

Supplementary Figure Legends

Supplementary Table

Supplementary Materials and Methods
