# Supplementary figures and images for: MiR-22 suppresses epithelial–mesenchymal transition in bladder cancer by inhibiting Snail and MAPK1/Slug/vimentin feedback loop
Source: Cell Death Dis. 2018 Feb 12;9(2):209. doi: 10.1038/s41419-017-0206-1 (PMC5833802; doi:10.1038/s41419-017-0206-1)

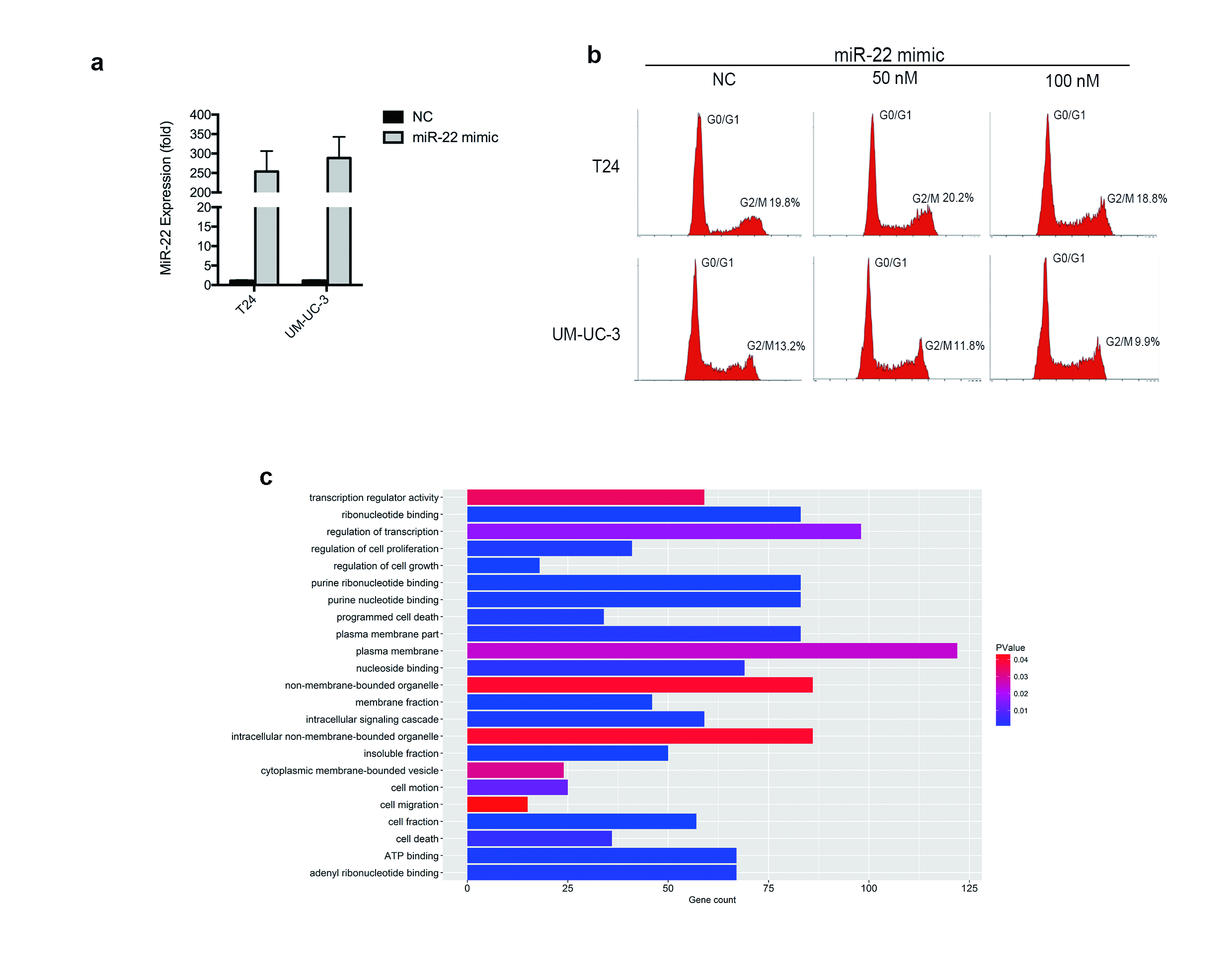

Supplement: Supplementary file 1 — Supplementary Figure 1 [file 41419_2017_206_MOESM1_ESM.tif]

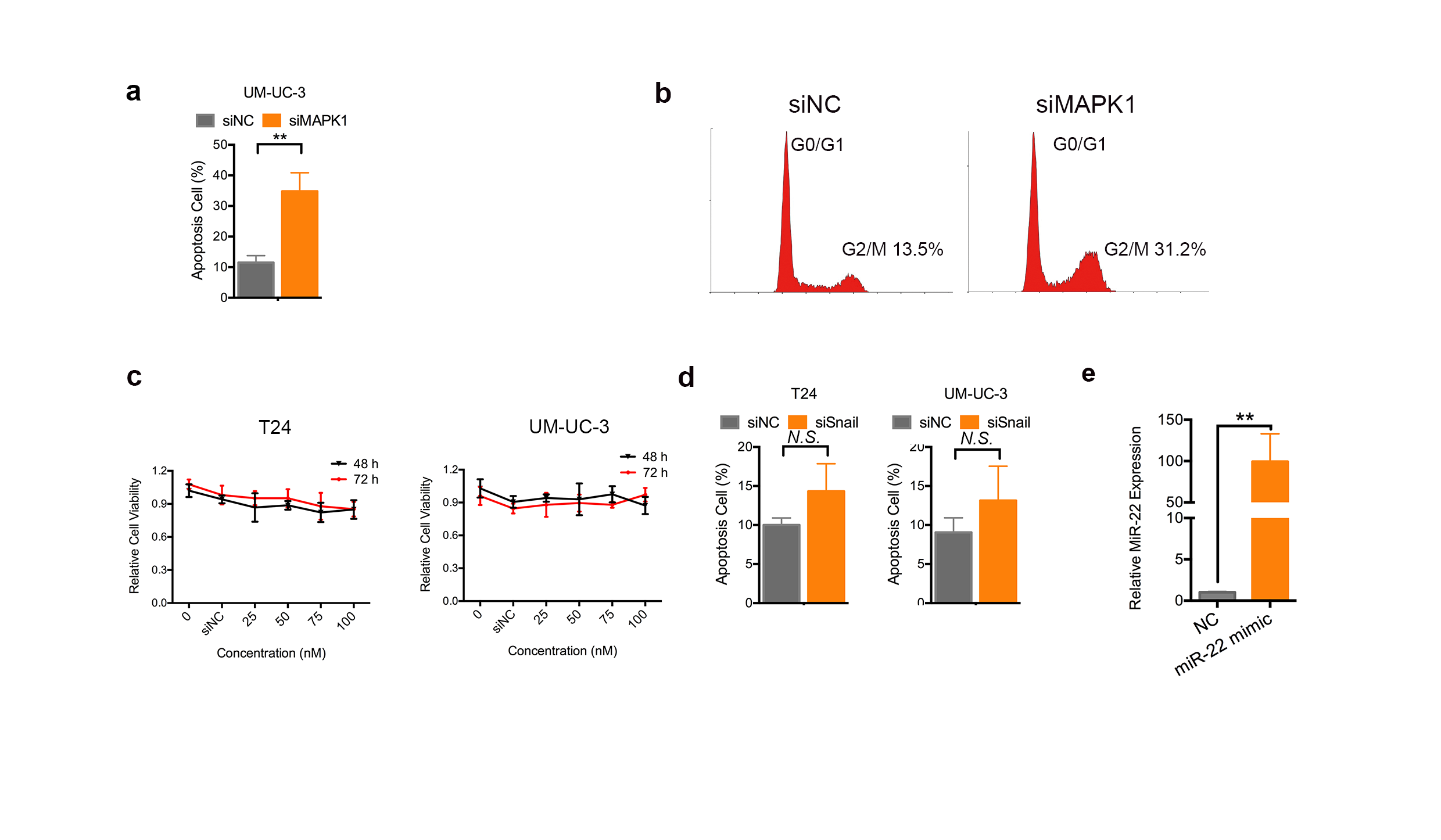

Supplement: Supplementary file 2 — Supplementary Figure 2 [file 41419_2017_206_MOESM2_ESM.tif]
