# Supplementary material for: MiR-22 suppresses epithelial–mesenchymal transition in bladder cancer by inhibiting Snail and MAPK1/Slug/vimentin feedback loop
Source: Cell Death Dis. 2018 Feb 12;9(2):209. doi: 10.1038/s41419-017-0206-1 (PMC5833802; doi:10.1038/s41419-017-0206-1)
Supplement: Supplementary file 3 — Supplementary Figure Legends [file 41419_2017_206_MOESM3_ESM.docx]

**Supplementary Figure Legends**

**Supplementary Figure 1.** (**a**) qRT-PCR analysis. BCa cells were treated with 50 nM miR-22 mimics or mimic negative control (NC) for 48 h. An increase in the expression of miR-22 was detected in miR-22 mimic transfected BCa cells. (**b**) Representative images of cell cycle assay. BCa cells were treated with miR-22 mimics or NC for 48 h. Cell cycle DNA content in G0/G1, G2/M phase was evaluated by flow cytometry analysis. Overexpression of miR-22 had little influence on cell cycle of BCa cells. (**c**) KEGG analysis indicated that 540 miR-22 potential targets were involved in cancer and cell motility-related pathways. Error bars represent the S.D. obtained from three independent experiments. **P* < 0.05, ***P* < 0.01, *** *P* < 0.001.

**Supplementary Figure 2.** (**a**) Apoptosis analysis. UM-UC-3 cells were treated with 50 nM small interfering RNA targeting human MAPK1 mRNA (named siMAPK1) or siRNA negative control (siNC) for 48 h. The results showed that silencing of MAPK1 effectively induced UM-UC-3 cell apoptosis. (**b**) Representative images of cell cycle assay. T24 cells were treated with siMAPK1 or siNC for 48 h, then cell cycle DNA content in G0/G1, G2/M phase was evaluated by flow cytometry analysis. The results showed that knockdown of MAPK1 effectively arrested the cell cycle of BCa cells. (**c**) Cell count kit-8 (CCK-8) assay. BCa cells were treated with 50 nM small interfering RNA targeting human Snail mRNA (named siSnail) or siNC for 48 h. Silencing of Snail had little influence on the proliferation of BCa cells. (**d**) Apoptosis assay. BCa cells were treated as (**c**). The results showed that silencing of Snail had little influence on BCa cell apoptosis. (**e**) qRT-PCR assay of tumor tissue. UM-UC-3 cells that pre-transfected with miR-22 mimic or nonsense dsRNA (NC) were implanted into nude mice. The miR-22 expression levels were higher detected in the tumor tissues from miR-22 treated group compared with that in the tumor tissues from NC treated group. Error bars represent the S.D. obtained from three independent experiments. **P* < 0.05, ***P* < 0.01, *** *P* < 0.001.
