# Supplementary material for: MiR-22 suppresses epithelial–mesenchymal transition in bladder cancer by inhibiting Snail and MAPK1/Slug/vimentin feedback loop
Source: Cell Death Dis. 2018 Feb 12;9(2):209. doi: 10.1038/s41419-017-0206-1 (PMC5833802; doi:10.1038/s41419-017-0206-1)
Supplement: Supplementary file 4 — Supplementary Table [file 41419_2017_206_MOESM4_ESM.docx]

Supplementary Table 1: Patient Information

| Patient  Number | Sex | Age | TNM Stage | Histological Grade |
| --- | --- | --- | --- | --- |
| 1 | M | 62 | TNM II | III |
| 2 | M | 60 | TNM I | I |
| 3 | M | 53 | TNM I | III |
| 4 | M | 86 | TNM I | III |
| 5 | M | 55 | TNM I | II |
| 6 | F | 74 | TNM II | III |
| 7 | M | 56 | TNM II | III |
| 8 | F | 76 | TNM III | III |
| 9 | M | 65 | TNM II | II |
| 10 | F | 69 | TNM II | II |
| 11 | M | 72 | TNM III | III |
| 12 | M | 78 | TNM I | II |
| 13 | M | 76 | TNM III | III |

Abbreviations: TMN, Tumor Node Metastasis；M, Male; F, Female

Supplementary Table 2: Patient Information

|  | **TNM Stage** | | | |
| --- | --- | --- | --- | --- |
| **Characteristic** | I  (n = 2) | II  (n =128) | III  (n = 139) | IV  (n = 132) |
| **Age—yr** |  |  |  |  |
| Median | 61.5 | 66.5 | 69 | 69 |
| Age group—n (%) |  |  |  |  |
| 30-59 yr | 1 (50.0) | 39 (30.5) | 29 (20.9) | 19 (14.4) |
| 60-74 yr | 1 (50.0) | 56 (43.8) | 66 (47.5) | 63 (47.7) |
| 75 yr or older | 0 (0.0) | 33 (25.8) | 44 (31.7) | 50 (37.9) |
| **Sex—n (%)** |  |  |  |  |
| Male (n = 296) | 2 (100.0) | 96 (75.0) | 101 (72.7) | 97 (73.5) |
| Female (n =105) | 0 (0.0) | 32 (25.0) | 38 (27.3) | 35 (26.5) |

Abbreviations: yr, year; n, number of patients; %, percentage; TNM, Tumor Node Metastasis；

Supplementary Table 3: Primers for mRNA quantitative real-time PCR

| **Primer** | **Sequence (5’-3’)** |
| --- | --- |
| MAPK1-F | TACACCAACCTCTCGTACATCG |
| MAPK1-R | CATGTCTGAAGCGCAGTAAGATT |
| Snail-F | TCGGAAGCCTAACTACAGCGA |
| Snail-R | AGATGAGCATTGGCAGCGAG |
| Slug-F | CGAACTGGACACACATACAGTG |
| Slug-R | CTGAGGATCTCTGGTTGTGGT |
| Vimentin-F | GACGCCATCAACACCGAGTT |
| Vimentin-R | CTTTGTCGTTGGTTAGCTGGT |
| N-cadherin-F | TTTGATGGAGGTCTCCTAACACC |
| N-cadherin-R | ACGTTTAACACGTTGGAAATGTG |
| E-cadherin-F | CGAGAGCTACACGTTCACGG |
| E-cadherin-R | \| GGGTGTCGAGGGAAAAATAGG \|  \| \| --- \| --- \| |
| GSK-3βF | GGCAGCATGAAAGTTAGCAGA |
| GSK-3β-R | GGCGACCAGTTCTCCTGAATC |
| GAPDH-F | GGAGCGAGATCCCTCCAAAAT |
| GAPDH-R | GGCTGTTGTCATACTTCTCATGG |

Supplementary Table 4: siRNAs used in this study

| **siRNA** | **Sequence (5’-3’)** |
| --- | --- |
| Snail-sense  Snail-antisense | CCGGGCAAUUUAACAAUGUUU |
|  | UUGGCCCGUUAAAUUGUUACA |
| MAPK1-sense  MAPK1-antisense | GCAGGAGCUUGUGGAAAUAUU |
|  | UUCGUCCUCGAACACCUUUAU |
| Slug-sense  Slug-antisense | GGUCGUAAUACACCUAAAUUU |
|  | UUCCAGCAUUAUGUGGAUUUA |
| Vimentin-sense  Vimentin-antisense | GCAGAAGAAUGGUACAAAUUU |
|  | UUCGUCUUCUUACCAUGUUUA |
