# Supplementary material for: MiR-22 suppresses epithelial–mesenchymal transition in bladder cancer by inhibiting Snail and MAPK1/Slug/vimentin feedback loop
Source: Cell Death Dis. 2018 Feb 12;9(2):209. doi: 10.1038/s41419-017-0206-1 (PMC5833802; doi:10.1038/s41419-017-0206-1)
Supplement: Supplementary file 5 — Supplementary Materials and Methods [file 41419_2017_206_MOESM5_ESM.docx]

**Supplementary materials and methods**

**Immunohistochemical staining (IHC) analysis.** Antibodies specific for Snail (ab53519), MAPK1 (ab32081) and Ki67 (ab15580) were purchased from Abcam (MA, USA) and used for the IHC analysis of paraffin-embedded sections of animal experiments. The 4-μm sections were cut, deparaffinized and rehydrated. Endogenous peroxidases were blocked in 0.3% hydrogen-peroxide methanol for 20 min. Heat-induced antigen retrieval was conducted for 15 min in a microwave oven. Sections were incubated overnight with primary antibodies using predetermined optimal dilutions and incubation times. The slides were then incubated with the goat anti-rabbit IgG/HRP complex (ZSGB-BIO, PV-6001, Beijing, China) for 45 min at room temperature. After washing the slides two times in PBS, the sections were incubated with horseradish peroxidase-conjugated streptavidin (DAB kit, ZSGB-BIO, China) for 30 min and then washed twice in PBS. The positive results were visualized with 3,3’-diaminobenzidine (DAB) (DAB kit, ZSGB-BIO, China). The results of IHC were analyzed by examination and photography under microscopy with × 400 magnification.

**Annexin V and propidium iodide staining analysis.** BCa cells with different treatments were collected and subjected to Annexin V and propidium iodide staining using Annexin V-FITC/PI apoptosis kit (Multi Science, AP101, China) according to the manufacturer’s protocol. After staining, flow cytometry was performed to quantify apoptotic cells.

**Flow cytometry analysis of cell cycle.** BCa cells were fixed in −20 °C absolute ethanol for 4 h and resuspended in 3 ml of PBS. After incubating at 37 ^o^C for 30 min, the cells were treated with DNA staining solution (Multi Science, CCS012) at room temperature for 10 min in the dark. A total of 10,000 cells were analyzed by flow cytometry.

**RNA extraction and real-time quantitative PCR (qRT-PCR) analysis.** Total RNA from BC cell lines was extracted using RNAiso Plus (TaKaRa, 9109, Kusatsu, Japan) following the manufacturer’s instructions. Total RNA from the paraffin-embedded sections of animal experiments was extracted using the RNeasy^®^FFPE Kit (Invitrogen, 73504, Carlsbad, CA).

For mRNA qRT-PCR analysis, the total RNA was then reverse transcribed into cDNA (TaKaRa, R0037A) by random primers. Gene expression was measured using a LightCycler 480II system (Roche Diagnostics, Basel, Switzerland) with SYBR Premix EX Tag kit (TaKaRa, RR420A) and specific primers (Supplementary Table 3). The miRNA quantification was determined by Bulge-loop^TM^ miRNA qRT-PCR Primer Set (one RT primer and a pair of qPCR primers for each set) specific for miR-22 and U6, designed by RiboBio. Relative RNA expression was calculated with the 2 –ΔΔCt method after normalizing the expression levels of tested mRNA to GAPDH mRNA and tested miRNA to U6 miRNA.

**Western blotting assay.** Antibodies specific to phospho-ERK1/2 (4379), ERK1/2 (9102), vimentin (5741), N-cadherin (13116), E-cadherin (3195), Snail (3879), Slug (9585), GSK-3β (5558), phospho-GSK-3β (9323) and GAPDH (5714) were purchased from Cell Signaling Technology (CST, Beverly, MA). The cell lysates were collected with radioimmunoprecipitation assay (RIPA) lysis buffer (Beyotime Biotechnology) and 1 mM phenylmethanesulfonyl fluoride (PMSF; Beyotime Biotechnology). The protein concentration was assessed using a BCA assay (BCA Protein Assay Kit, Beyotime, P0012). Denatured protein lysates (50 μg, 70°C for 10 min) were separated on 10% SDS-polyacrylamide gels and transferred to PVDF membranes (0.2 mm, Millipore, Darmstadt, Germany). Western blotting was performed according to the manufacturer’s instructions. Membranes were blocked in Tris-buffered saline containing 0.05% Tween-20 (Sangon Biotech) and 5% non-fat dry milk for 1 h. After blocking, the membranes were probed overnight at 4°C with primary antibodies. Then, the blots were incubated for 2 h at room temperature in a 1:10,000 dilution of HRP goat anti-rabbit IgG antibody (Abcam, ab6721) after 3 washes with PBST. After extensive washing, antibody detection was accomplished with a sensitive substrate (Immun-Star^TM^ Western C^TM^ Kit, Bio-Rad).

**Statistical analysis.** Statistical analyses were performed with GraphPad Prism software (GraphPad, San Diego, CA, USA). Briefly, the data are presented as the mean ± standard error deviation (S.D.) of three independent experiments. Two-way analysis of variance (ANOVA) followed by Bonferroni’s posttest was used to compare the means of groups influenced by two independent factors, whereas one-way ANOVA followed by Tukey’s posttest was used to compare the means of three independent groups. Student’s *t*-test followed by Welch’s correction was used to compare the means of two independent groups. The correlation between two factors was evaluated by correlation analysis, and Spearman correlation coefficients were calculated to estimate the correlations. Statistical evaluation of mRNA expression levels in clinical BCa tissues was performed with the Kruskal-Wallis test with Dunn’s correction for multiple testing. The Kaplan-Meier survival function was calculated and compared with a log-rank test to assess the differences in BCa samples. *P* values < 0.05 were considered statistically significant.

**Ethics statement.** All studies that involved human participants were approved by the Ethics Committee of Zhejiang University School of Medicine, Hangzhou, China, and the study methodology was carried out in accordance with the approved guidelines. Informed consent was obtained from each subject before participating in the study, according to the Declaration of Helsinki. All of the animal experiments in this study were approved by the Zhejiang University Animal Care Committee, Hangzhou,China. All animal manipulations were carried out according to the National Institutes of Health Guidelines for the Care and Use of Laboratory Animals (NIH Publication, 8^th^ edition) as revised in 2012. The mice were sacrificed using carbon dioxide inhalation. All surgeries were conducted under sodium pentobarbital anesthesia, and all efforts were made by the attending skilled technician to minimize suffering.
